# Adherence to the International Committee of Medical Journal Editors’ (ICMJE) prospective registration policy and implications for outcome integrity: a cross-sectional analysis of trials published in high-impact specialty society journals

**DOI:** 10.1186/s13063-018-2825-y

**Published:** 2018-08-23

**Authors:** Anand D. Gopal, Joshua D. Wallach, Jenerius A. Aminawung, Gregg Gonsalves, Rafael Dal-Ré, Jennifer E. Miller, Joseph S. Ross

**Affiliations:** 1 Yale University School of Medicine, Harkness Hall, 367 Cedar Street, Box #415, New Haven, CT 06510 USA; 2 Center for Outcomes Research and Evaluation (CORE), Yale-New Haven Hospital and Collaboration for Research Integrity and Transparency, Yale University, New Haven, CT USA; 3 Yale School of Public Health, New Haven, CT USA; 4 Epidemiology Unit, Health Research Institute-Fundación Jiménez Díaz University Hospital, Universidad Autónoma de Madrid, Madrid, Spain; 5 Division of Medical Ethics, Department of Population Health, NYU School of Medicine, New York, NY USA; 6 Bioethics International, New York, NY USA; 7 Department of Internal Medicine, Yale University School of Medicine, New Haven, CT USA

**Keywords:** Trial registration, ICMJE, Selective reporting

## Abstract

**Background:**

Registration of clinical trials is critical for promoting transparency and integrity in medical research; however, trials must be registered in a prospective fashion to deter unaccounted protocol modifications or selection of alternate outcomes that may enhance favorability of reported findings. We assessed adherence to the International Committee of Medical Journal Editors’ (ICMJE) prospective registration policy and identified the frequency of registrations occurring after potential observation of primary outcome data among trials published in the highest-impact journals associated with US professional medical societies. Additionally, we examined whether trials that are unregistered or registered after potential observation of primary outcome data were more likely to report favorable findings.

**Methods:**

We conducted a retrospective, cross-sectional analysis of the 50 most recently published clinical trials that reported primary results in each of the ten highest-impact US medical specialty society journals between 1 January 2010 and 31 December 2015. We used descriptive statistics to characterize the proportions of trials that were: registered; registered retrospectively; registered retrospectively potentially after initial ascertainment of primary outcomes; and reporting favorable findings, overall and stratified by journal and trial characteristics. Chi-squared analyses were performed to assess differences in registration by journal and trial characteristics.

**Results:**

We reviewed 6869 original research reports published between 1 January 2010 and 31 December 2015 to identify a total of 486 trials across 472 publications. Of these 486 trials, 47 (10%) were unregistered. Among 439 registered trials, 340 (77%) were registered prospectively and 99 (23%) retrospectively. Sixty-seven (68%) of these 99 retrospectively registered trials, or 15% of all 439 registered trials, were registered after potential observation of primary outcome data ascertained among participants enrolled at inception. Industry-funded trials, those with enrollment sites in the US, as well as those assessing FDA-regulated interventions each had lower rates of retrospective registration. Unregistered trials were more likely to report favorable findings than were registered trials (89% vs. 64%; relative risk (RR) = 1.38, 95% confidence interval (CI) = 1.20–1.58; *p* = 0.004), irrespective of registration timing.

**Conclusions:**

Adherence to the ICMJE’s prospective registration policy remains sub-standard, even in the highest-impact journals associated with US professional medical societies. These journals frequently published unregistered trials and trials registered after potential observation of primary outcome data.

**Electronic supplementary material:**

The online version of this article (10.1186/s13063-018-2825-y) contains supplementary material, which is available to authorized users.

## Background

Registration of clinical trials is critical for promoting transparency and integrity in medical research, helping to ensure a complete and unbiased record of all clinical trials [1–3]. Registration alone, however, is insufficient, as trials must be registered in a prospective fashion to help mitigate selective reporting, which may include addition or removal of outcome measures, preferential publication of statistically significant findings, and modification of which outcome measures were pre-specified as primary [4].

The International Committee of Medical Journal Editors (ICMJE) adopted a policy to encourage prospective trial registration, mandating that all clinical trials beginning July 2005 register prospectively, at or before the time of first patient enrollment, as a condition for publication in its member journals [5]. Since implementation of this policy, trial registration at ClinicalTrials.gov, the largest international clinical trial registry, and other trial registries has increased substantially [6]. However, a small but notable number of trials remain unregistered, including those that are published [7–13].

Moreover, despite increasing rates of registration, prospective registration of trials is still lacking [7, 8, 11–16]. Although more than 2900 journals support general ICMJE manuscript publication guidelines [17], a 2014 survey found that journal editors do not consistently adhere to ICMJE’s prospective trial registration policy [18, 19]. Previous research suggests that even in the highest-impact general medical journals, 28% of published trials were registered retrospectively [15]. In some cases, registration occurred late enough to raise concerns about whether the specified primary outcome measure had been modified after trial inception [15], as retrospective registration may provide opportunity for unaccounted protocol modifications or selection of alternate outcomes to enhance the favorability of reported findings.

While previous studies of journal adherence to the ICMJE prospective trial registration policy have thus far either focused on the highest-impact general medical journals or sampled within field-specific journals [7, 8, 11–15], little is known about registration of trials published among high-impact specialty society journals. Specialty society journals, administered by professional organizations (e.g., the American Society of Clinical Oncology), tend to represent the views of their constituent specialists. They publish trials that are of great interest to their respective communities, which influence clinical practice [20]. Although specialty journals typically have lower impact factors than the highest-impact general medical journals, they are often a preferred source of clinical information and guidelines for specialists [21].

To assess trial registration within specialty society journal publications, we constructed a large sample of clinical trials recently published in ten high-impact US specialty society medical journals. We evaluated adherence to ICMJE prospective trial registration policy; identified instances of registration after potential observation of primary outcome data; and determined characteristics associated with prospective registration. We additionally compared registered and published primary outcomes among prospectively and retrospectively registered trials and identified predictors of primary outcome non-discordance. Finally, we examined whether trials that were unregistered or registered after potential observation of primary outcome data were more likely to report favorable study findings.

## Methods

See Additional file 1 for the study protocol.

### Journal selection

We identified US specialty society medical journals using a list of US-based medical professional organizations associated with any of the specialties registered with the American Board of Medical Specialties [22]. We searched for additional journals using SCImago Journal & Country Rank listings, adding to our list any journals associated with a US-based medical specialty organization [23]. We selected the ten journals with the highest-impact factors after excluding general practice journals and journals that do not publish clinical trials [24]. For each journal in our sample, we verified endorsement of trial registration as indicated by a statement on the journal’s website or listing of the journal on the ICMJE’s catalog of journals that follow its recommendations as a condition for inclusion [17].

### Clinical trial sample selection

We reviewed original research articles, including brief reports and communications but not research letters or correspondences, to identify the 50 most recent primary publications of clinical trials in each journal, beginning with articles published in print journal issues in December 2015 and continuing in reverse chronology as far back as January 2010. We used the table of contents of each print journal issue to identify articles for possible inclusion. Clinical trials were systematically identified by screening the article’s abstract and, if necessary, the Methods section, for statements meeting the World Health Organization’s (WHO) definition of a clinical trial, also used by ICMJE, namely any study that “prospectively assigns people or a group of people to an intervention, with or without concurrent comparison or control groups, to study the cause-and-effect relationship between a health-related intervention and a health outcome” [25].

We limited our sample to publications reporting the findings of a trial’s primary outcome(s), which is most pertinent to the information contained within its registration, and excluded trials reporting secondary analyses of previously published results, secondary outcomes only, or interim analyses of primary outcomes. We further excluded publications describing phase I trials, as these studies typically do not assess effectiveness and have minimal impact, if any, on clinical practice. We additionally excluded trials beginning prior to July 2005, since trials preceding the ICMJE policy were unlikely to be prospectively registered.

### Data collection

From trial publications, we extracted information on journal, intervention, allocation, manuscript submission date, enrollment start date, primary outcome(s) with associated findings, and registration number(s) corresponding to the trial(s) reported. To account for the possibility of duplicate registrations, we searched the WHO International Clinical Trials Registry Platform (ICTRP), which aggregates registrations across registries endorsed by WHO, and in turn endorsed by ICMJE, using the reported registration numbers and additionally reviewed registrations for alternate identifiers to determine the earliest registration for each trial. For publications not reporting registration information, we searched the WHO ICTRP platform using search terms pertaining to intervention, first author, senior author, and sponsor to identify unpublished registrations, cross-referencing potential matches against sample size and enrollment criteria. We contacted corresponding authors of unmatched trials for registration information before concluding that the published trial was unregistered.

Using the earliest registration for each trial, we collected registration date, primary outcome submission date, primary completion date, start date, primary outcome(s) at initial registration, enrollment, phase, location, and funding source. We supplemented information on the latter four elements from trial publications when missing from the registry. Among trials we determined to have been first registered at ClinicalTrials.gov, we additionally collected date of original primary outcome submission, which the registry uniquely lists separately from the trial registration date. We categorized intervention, funding source, location, enrollment, and allocation as outlined in Table 1 for use in pre-specified stratified analyses. We considered interventions involving drugs, devices, or biologicals as regulated by the Food and Drug Administration (FDA).

**Table 1:** Characteristics of clinical trials published in the 10 highest-impact US medical specialty society journals between 1 January 2010 and 31 December 2015 (*N* = 486)

	Number	Percentage
Intervention^a^		
Drug	287	59.1
Device	46	9.5
Vaccine or biological	86	17.7
Other	102	21.0
Phase^b^		
Phase II	190	39.1
Phase III	110	22.6
Phase IV	46	9.5
Not listed	153	31.5
Randomization		
Yes	372	76.5
No	114	23.5
Funding^c, d^		
Industry	216	44.4
Non-industry	270	55.6
Enrollment		
≥ 100	280	57.6
< 100	206	42.4
Location(s)		
US only	166	34.2
US and international	84	17.3
International only	236	48.6
Trial registry^e^		
ClinicalTrials.gov	383	87.2
EU-CTR	76	17.3
ISRCTN	24	5.5
Other registries^f^	47	10.7

^a^Clinical trials may have involved more than one intervention type

^b^13 trials were designated as phase II/phase III

^c^Funding information was not reported in the publications of 5 trials, all of which were unregistered; these trials were designated as not reporting industry funding

^d^Industry funding includes partial or full support

^e^439 trials were registered. Percentages are expressed based on a denominator of 439. 81 trials were registered in multiple registries, hence percentages may not sum to 100

^f^“Other registries” includes: Australia New Zealand Clinical Trials Register (ANZCTR), Chinese Clinical Trial Registry (ChiCTR), Clinical Trial Registry of India (CTRI), German Clinical Trials Register (DRKS), Japan Pharmaceutical Information Center Clinical Trials Information (JAPIC-CTI), Netherlands Trial Register (NTR), University Hospital Medical Information Network (UMIN) (a Japanese registry)

Abbreviations: *EU-CTR* European Union Clinical Trials Register, *ISRCTN* International Standard Randomized Controlled Trial Network

Data abstractions were performed in tandem by ADG and JAA. Consistency and accuracy were verified through a 10% random sample validation of each investigator’s collections. A third author (JDW) repeated all searches for trials that were determined to be unregistered, supplementing with additional searches of the National Institutes of Health (NIH) funding database using grant funding identifiers when listed in publications [26]. All disagreements were resolved by consensus with input from the senior investigator (JSR).

### Main outcome measures

We first determined whether each trial was registered by ensuring that a corresponding registration record could be located. For registered trials, we next ascertained timeliness of registration by determining whether the trial was registered within 30 days of its enrollment start date. Trials registered greater than 30 days after enrollment initiation were categorized as “retrospective.” Although ICMJE policy mandates registration at or before the time of first patient enrollment, we allowed a 30-day grace period between registration and enrollment initiation in order to account for potential flexibility on the part of journal editors with regard to registration timeliness. Further, because the FDA Amendments Act (FDAAA) of 2007 specifically mandates registration within 21 days of first patient enrollment as a legal mandate for studies of therapeutics and devices regulated by FDA, a 30-day window grace period was chosen to accommodate inconsistencies between legal and journal requirements. Month-based representations of dates were recorded as the last day of the corresponding month (i.e., September 2012 was transcribed as 30 September 2012) to conservatively classify registrations as “retrospective.” We elected to use enrollment start dates reported in registries as opposed to those reported in publications in our determinations of registration timeliness, as we believed that enrollment start date may not be consistently reported in publications, while, in registries, it is a mandatory registration element and, hence, less easily excluded or otherwise misrepresented.

Among trials registered retrospectively, we established whether registration might have occurred after initial ascertainment of the primary outcome, and hence potentially permitted unaccounted protocol modifications after interim analyses, by comparing the trial’s registration date against the date on which the primary outcome would have been collected for the trial’s first enrolled participant(s). For example, a trial with a primary outcome assessing serum creatinine levels at 6 weeks that registered in November 2012 but that began enrolling patients in February 2012 would have been retrospectively registered after observation of the primary outcome among participants enrolled at the trial’s initiation. In this case, investigators could have in theory observed primary outcome data for participants that enrolled at trial initiation and modified the trial based on data collected from the trial’s first participants. Without information on trial-specific recruitment, however, we cannot comment on how robust or useful this initial primary outcome data may have been in an interim analysis informing post hoc modifications, though we can comment on the theoretical potential for an interim analysis to have occurred in this scenario. In instances where multiple time frames were designated as primary, we based our calculations on the shortest primary outcome time frame specified in the registry. If no time frame was listed in association with the registered primary outcome, we used the time frame described in the trial’s publication. We noted cases where the nature of the primary outcome (e.g., median survival) did not permit this determination.

We next compared primary outcomes at initial registration against those specified in publications, excluding any primary outcomes pertaining specifically to safety or tolerability. We classified primary outcomes as discordant or non-discordant. Primary outcome comparisons were performed in a systematic manner across three sequential domains: number of primary outcome(s), definition(s) of primary outcomes, and specified time frame(s) for primary outcome(s) ascertainment. Specifically, we began by first comparing the number of primary outcomes in the initial registration record against the number of outcomes explicitly stated as primary in the publication. If the number of primary outcomes differed, the primary outcomes were classified as discordant. If the number of primary outcomes was the same, we compared the definition of the primary outcome (e.g., all-cause mortality vs. recurrent VTE). If the primary outcomes differed in how they were defined, they were classified as discordant. Because primary outcomes may be registered in varying levels of detail, it may not be possible to know with certainty whether registered and published primary outcomes are truly concordant. Accordingly, we classified registered and published primary outcome definitions as discordant on the basis of explicit known differences in what was specified, rather than focusing on differences in level of specification. For example, if a trial registration specified a primary outcome definition of “anxiety level” and the publication reported a primary outcome definition of “Hamilton Anxiety Rating Scale,” we classified these outcome definitions as non-discordant rather than discordant. In not doing so, we sought to avoid classifying primary outcomes as discordant based simply on differences in the level of detail provided between registrations and published reports. In cases where the number of registered and published primary outcomes was the same but greater than one, registered and published primary outcome definitions were paired and compared on the basis of clinical judgment with input from the senior investigator. If primary outcome definitions did not differ, we next compared the time frame, if available, at which the primary endpoints were ascertained. If the explicitly stated time frames differed (i.e., 3-month mortality vs. 30-day mortality), these outcomes were classified as discordant. We noted cases where registered outcomes were too poorly specified (e.g., vague study of “efficacy of intervention”) to permit comparison. Trials without a designated primary outcome specified in the publication were excluded from outcome comparison analyses.

Finally, we categorized each trial on the basis of its primary outcome findings whenever formal hypothesis testing had been conducted or inferences could be made regarding the statistical significance of reported findings (i.e., inferential studies). By examining trial publications, we determined whether the trial’s findings indicated, based on the reported primary outcome(s), that a study intervention was statistically significantly better (i.e., positive), statistically significantly worse (i.e., negative), or not statistically significantly different (i.e., not significant) than a designated comparison (placebo group, active group, or predefined threshold) and classified the overall trial accordingly. For trials that assessed a non-inferiority hypothesis, we considered establishment of non-inferiority to represent a “positive” result and failure to establish non-inferiority a “not significant” result. In instances where more than one primary outcome was reported, we categorized trials with at least one significant primary outcome as “positive” or “negative” on the basis of the statistically significant outcome; trials with mixed findings (some positive and some negative primary outcomes) were classified by prioritizing the results of clinical outcomes over surrogate markers. Trials with mixed all clinical or all surrogate primary outcome results were arbitrated based on the relative importance of the significant outcomes in question. For trials that did not specifically designate a primary outcome, any outcomes reported in the trial’s abstract were considered primary and the overall study was categorized using the scheme described previously. Trials that presented analyses in a solely descriptive manner or that lacked a designated comparison against which to judge the statistical significance of reported results were noted as “non-inferential” and excluded from analyses of association. Trials categorized as “positive” were judged to report overall favorable findings, whereas those categorized as “negative” or “not significant” were judged to report overall unfavorable findings.

### Statistical analysis

We used descriptive statistics to characterize the proportion of trials registered, the proportion registered retrospectively, as well as the proportion registered after initial primary outcome ascertainment, overall, and stratified by specialty journal and trial characteristics. We additionally determined the proportion of trials with non-discordant registered and published primary outcome measures and the proportion reporting favorable findings, overall, and stratified by journal, registration timeliness, intervention, funding source, location, allocation, and enrollment. We used chi-squared testing to assess differences in registration and registration timeliness by journal and by each of the aforementioned trial characteristics. We also used chi-squared testing to assess differences in primary outcome concordance and study findings by journal, trial characteristics, and timeliness of registration. All statistical hypothesis testing was performed using chi-squared analyses with a two-sided type I error level of 0.006 to account for multiple comparisons. Accordingly, all reported *p* values correspond to chi-squared testing. We additionally report relative risks (RR) with 95% confidence intervals (CI) for all chi-squared comparisons involving two binary variables. Statistical analyses were performed using JMP Pro Version 11.2.0 (SAS Institute, Inc.).

## Results

### Search results

**Fig. 1:**
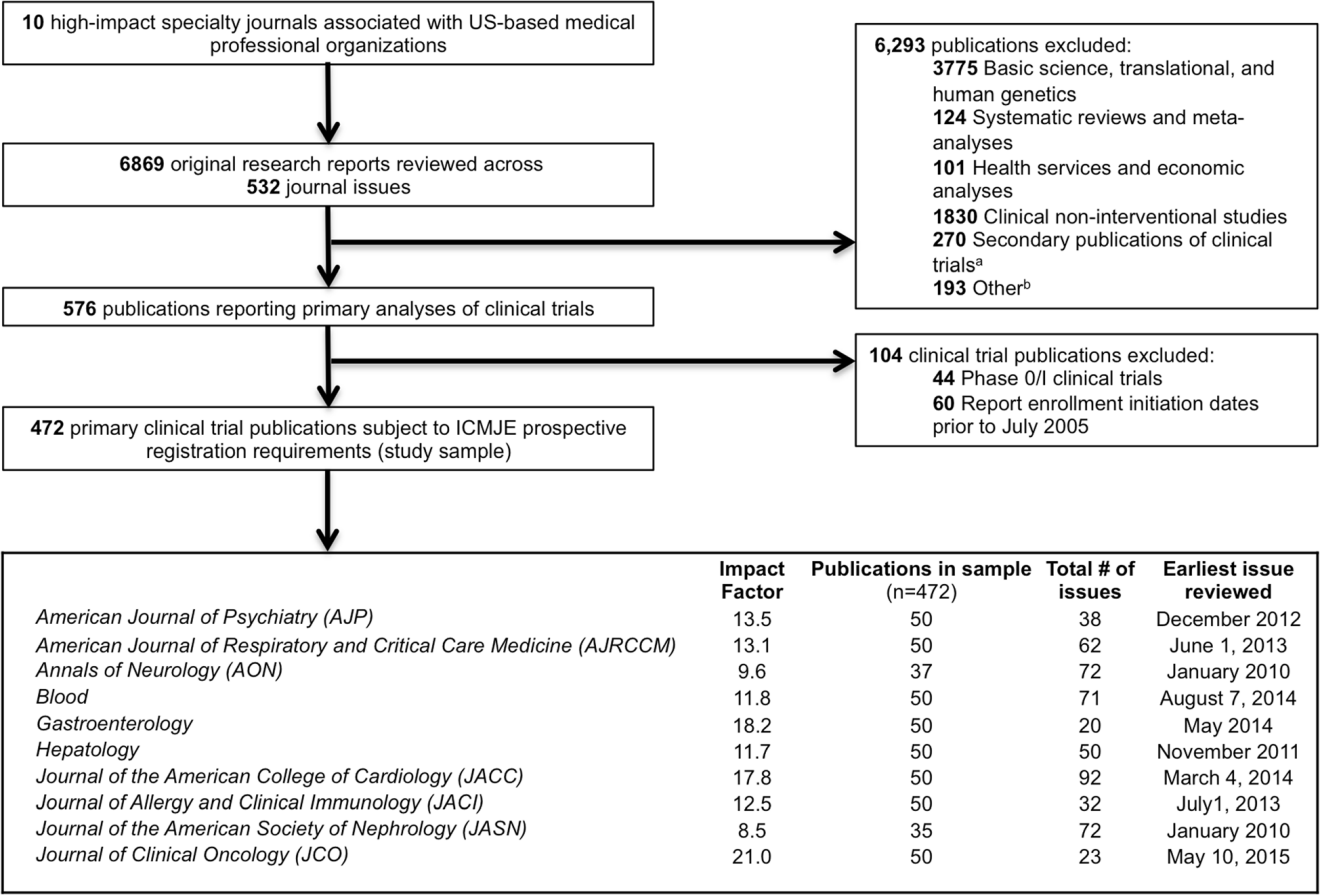
Construction of study sample comprising the 50 most recent clinical trial publications appearing in each of ten high-impact US medical specialty society journals between 1 January 2010 and 31 December 2015. ^a^Includes post hoc analyses, exploratory analyses, analyses of secondary outcomes, long-term follow-up, interim analyses, pooled analyses, extension trials, and studies utilizing data derived from clinical trials. ^b^Includes case reports, case series, modeling studies, and twin studies

We reviewed 6869 original research reports published in the period between 1 January 2010 and 31 December 2015 to identify the 50 most recent primary trial publications in each of ten high-impact specialty journals (Fig. 1). Two journals (*Annals of Neurology*, *n* = 37; *Journal of the American Society of Nephrology*, *n* = 35) published fewer than 50 eligible primary trial publications in this period. After excluding publications describing phase I trials (*n* = 44) and trials initiating enrollment prior to July 2005 (*n* = 60), there were 472 publications reporting the primary results of 486 clinical trials (14 articles described multiple trials).

### Characteristics of eligible trials

Among our final sample of 486 trials, 76% (*n* = 372) were randomized studies (Table 1). Eighty-one percent (*n* = 392) assessed interventions involving drugs, devices, or vaccines/biologicals. Forty-four percent received industry funding (*n* = 216), and just over half recruited patients at one or more sites located in the US (*n* = 250; 51%). Phase II designations were most frequent (*n* = 190; 39%). Median enrollment across all trials was 127 participants (interquartile range (IQR), 49–300). Eighty-nine percent (*n* = 433) of trials were published since 2013. The median impact factor among journals in our sample was 12.24 (range, 8.5–21.0).

### Registration

Forty-seven (10%) of the 486 trials were not registered. Two trials (0.4%) reported registration numbers for which a matching registration could not be located. Of 439 registered trials, 33 (8%) did not report a trial registration number in their publication, requiring further searching to identify corresponding registrations records. All registered trials (*n* = 439, 100%) were registered in registries endorsed by ICMJE, though duplicate registrations across more than one registry were not uncommon (*n* = 81; 18%). Eighty-seven % of registered trials (*n* = 383) were registered at ClinicalTrials.gov, which accounted for 79% of initial trial registrations (*n* = 346).

Specialty journals differed in their rates of trial registration (Table 2) (*p* < 0.001). *Annals of Neurology* published the greatest proportion of unregistered trials (43%; 16 of 37), accounting for 34% of trials without registration; in comparison, all trials published in the *Journal of Clinical Oncology* and *Blood*, both of which primarily publish oncology trials, were registered. Registration was more frequent among trials involving drugs, devices, or vaccines/biologicals (361 of 392; 92%) compared to those involving other intervention types (78 of 94; 83%), though this did not reach statistical significance (RR = 1.11, 95% CI = 1.01–1.22; *p* = 0.007). Randomization, larger trial size, enrollment sites in the US, and industry funding were each additionally associated with higher rates of registration (Table 3).

**Table 2:** Registration, timeliness of registration, and primary outcome non-discordance among clinical trials published in 10 high-impact US medical specialty society journals, stratified by journal

	Total (column %)	Registration		Timeliness of registration^a^				Primary outcome comparison^e^		Primary outcome results	
		Unregistered (%)^b^	Overall chi-squared *p* value	Retrospective (%)^c^	Overall chi-squared *p* value	Retrospective after initial primary outcome ascertainment (%)^c, d^	Overall chi-squared *p* value	Non-discordant (%)^c^	Overall chi-squared *p* value	Favorable (%)^f^	Overall chi-squared *p* value
Total	486 (100)	47 (9.7)	< 0.001	99 (22.6)	0.21	67 (15.3)	0.004	249 (56.7)	.049	282 (66.4)	0.11
*AJP*	52 (10.7)	6 (11.5)		15 (32.6)		14 (30.4)		17 (37.0)		45 (86.5)	
*AJRCCM*	51 (10.5)	4 (7.8)		7 (14.9)		7 (14.9)		28 (59.6)		29 (60.4)	
*AON*	37 (7.6)	16 (43.2)		5 (23.8)		3 (14.3)		8 (38.1)		24 (72.7)	
*Blood*	52 (10.7)	0 (0.0)		9 (17.3)		4 (7.7)		26 (50.0)		23 (60.5)	
*Gast.*	53 (10.9)	1 (1.9)		7 (13.4)		7 (13.5)		30 (57.7)		30 (68.2)	
*Hep.*	50 (10.3)	7 (14.0)		9 (20.9)		4 (9.3)		27 (62.8)		21 (60.0)	
*JACI*	51 (10.5)	7 (13.7)		12 (27.3)		5 (11.4)		25 (56.8)		27 (64.3)	
*JCO*	51 (10.5)	0 (0.0)		10 (19.6)		4 (7.8)		35 (68.6)		28 (57.1)	
*JACC*	50 (10.3)	4 (8.0)		16 (34.8)		15 (32.6)		34 (73.9)		30 (66.7)	
*JASN*	39 (8.0)	2 (5.1)		9 (24.3)		4 (10.8)		19 (51.4)		25 (64.1)	

^a^Among 439 registered trials, we could not determine timeliness of registration for 2 (1 published in *Gastroenterology* and the other in *JCO),* as enrollment start date was missing from registrations. We excluded these 2 trials from analyses of association pertaining to overall timeliness of registration and timeliness of registration relative to initial primary outcome ascertainment

^b^Percentages are expressed as fraction of total trials in each row

^c^Percentages are expressed as fraction of registered trials in each row

^d^Due to the nature of the primary outcome (i.e., median survival), we could not determine if retrospective registration occurred after initial primary outcome ascertainment in 8 cases: 1 in *Blood;* 1 in *Hepatology*; 2 in *JACI*; and 4 in *JCO*. These trials were excluded from analyses of association pertaining to timeliness of registration relative to initial primary outcome ascertainment

^e^26 of 439 registered trials did not have a primary outcome designated in their publication and were, therefore, excluded from analyses of association pertaining to primary outcome concordance

^f^Percentages are expressed as fraction of trials in each journal for which primary outcome favorability could be judged (row totals not shown)

Abbreviations: *AJP* American Journal of Psychiatry, *AJRCCM* American Journal of Respiratory and Critical Care Medicine, *AON* Annals of Neurology, *Gast.* Gastroenterology, *Hep.* Hepatology, *JACI* Journal of Allergy and Clinical Immunology, *JCO* Journal of Clinical Oncology, *JACC* Journal of the American College of Cardiology, *JASN* Journal of the American Society of Nephrology

**Table 3:** Registration, timeliness of registration, primary outcome concordance, and study results across clinical trials published in 10 high-impact US medical specialty society journals, stratified by trial characteristics

	Total (column %)	Registration		Timeliness of registration^c, d^				Primary outcome Comparison^g, h^		Primary outcome Results^i^	
		Unregistered (%)^b^	RR (95% CI); chi-squared *p* value	Retrospective (%)^e^	RR (95% CI); chi-squared *p* value	Retrospective after initial primary outcome ascertainment (%) ^e, f^	RR (95% CI); chi-squared *p* value	Non-discordant (%)^e^	RR (95% CI); chi-squared *p* value	Favorable (%)^j^	RR (95% CI); chi-squared *p* value
Total	486 (100)	47 (9.7)		99 (22.6)		67 (15.3)		249 (56.7)		282 (66.4)	
Drug/device/ biological											
Yes	392 (80.7)	31 (7.9)	0.46 (0.27–0.81); 0.0073	65 (18.0)	0.42 (0.30–0.58); < 0.001	42 (11.6)	0.37 (0.24–0.57); < 0.001	216 (59.8)	1.36 (1.05–1.77); 0.009	218 (64.7)	0.89 (0.77–1.03); 0.16
No	94 (19.3)	16 (17.0)		34 (43.6)		25 (32.1)		33 (42.3)		64 (72.7)	
Funding^a^											
Industry	216 (44.4)	11 (5.1)	0.38 (0.20–0.73); 0.0023	25 (12.2)	0.39 (0.26–0.58); < 0.001	18 (8.8)	0.41 (0.25–0.68); < 0.001	131 (63.9)	1.22 (1.04–1.42); 0.014	117 (65.7)	0.98 (0.86–1.13); 0.82
Non-industry	270 (55.6)	36 (13.3)		74 (31.6)		49 (20.9)		118 (50.4)		165 (66.8)	
Location											
≥ 1 US site	250 (51.4)	15 (6.0)	0.44 (0.25–0.80); 0.0048	35 (14.9)	0.47 (0.33–0.68); < 0.001	23 (9.8)	0.44 (0.28–0.71); < 0.001	133 (56.6)	0.94 (0.80–1.10); 0.44	148 (65.8)	0.98 (0.86–1.12); 0.79
Non-US	236 (48.6)	32 (13.5)		64 (31.4)		44 (21.6)		116 (56.9)		134 (67.0)	
Randomized											
Yes	372 (76.5)	23 (6.2)	0.29 (0.17–0.50); < 0.001	79 (22.6)	1.02 (0.66–1.58); 0.91	56 (16.0)	1.30 (0.71–2.36); 0.39	195 (55.9)	0.85 (0.72–1.02); 0.10	231 (64.0)	0.80 (0.69–0.93); 0.01
No	114 (23.5)	24 (21.1)		20 (22.2)		11 (12.2)		54 (60)		51 (79.7)	
Enrollment											
≥ 100	280 (57.6)	9 (3.2)	0.17 (0.09–0.35); < 0.001	58 (21.4)	0.88 (0.62–1.25); 0.49	43 (15.9)	1.10 (0.69–1.74); 0.69	162 (59.8)	1.06 (0.90–1.25); 0.47	161 (62.7)	0.87 (0.76–0.99); 0.046
< 100	206 (42.4)	38 (18.5)		41 (24.4)		24 (14.3)		87 (51.8)		121 (72.0)	

^a^Trials receiving either full or partial industry support were designated as having received industry funding

^b^Percentages are expressed as the fraction of total trials in each row

^c^Trials registered > 30 days after enrollment start were considered to have been registered retrospectively. Note that ICMJE policy mandates registration prior to enrollment start

^d^Among 439 registered trials, we could not determine timeliness of registration for 2 (1 published in *Gastroenterology* and the other in *Journal of Clinical Oncology)*, as enrollment start date was missing from registrations. We excluded these 2 trials from analyses of association pertaining to overall timeliness of registration and timelines of registration relative to initial primary outcome ascertainment

^e^Percentages are expressed as the fraction of registered trials (total −kindly advise if my minus sign inclusion here is correct; please alter as need if not unregistered) in each row

^f^Due to the nature of the primary outcome (i.e., median survival), we could not determine if retrospective registration occurred after initial primary outcome ascertainment in 8 cases: 1 in *Blood;* 1 in *Hepatology*; 2 in *Journal of Allergy and Clinical Immunology*; and 4 in *Journal of Clinical Oncology.* These trials were excluded from analyses of association pertaining to timeliness of registration relative to initial primary outcome ascertainment

^g^Registered and published primary outcomes were considered non-discordant if they did not explicitly differ in any of the following 3 domains: number of outcomes, outcome definition(s), or outcome time frame(s)

^h^26 of 439 registered trials did not have a primary outcome designated in their publication and were, therefore, excluded from analyses of association pertaining to primary outcome comparison

^i^Primary outcome favorability could not be judged for 61 trials. These trials were excluded from analyses of association pertaining to primary outcome favorability

^j^Percentages are expressed as the fraction of trials in each row for which primary outcome favorability could be judged (row totals not shown)

Abbreviations: *RR* relative risk, *CI* confidence interval

### Timeliness of registration

Among the 439 registered trials, 99 (23%) were registered retrospectively (i.e., at least 30 days after beginning patient enrollment) based on the enrollment start date reported in the registry. The median delay in registration was 8 months (IQR, 5–19; range, 1–88). Sixty-seven (68%) of the 99 retrospectively registered trials, or 15% of all 439 registered trials, were registered after potential observation of primary outcome data after collection of the primary outcome among participants enrolled at inception (Table 4). Of 302 trials with a registered primary completion date, 7 (2%) were registered after reported completion of data collection for the trial’s primary outcomes. Two (2%) of 88 retrospectively registered trials that listed a manuscript submission date were found to have registered after submission of the manuscript to the publishing journal, though this does not account for retrospective registrations that may have occurred after prior journal submissions to journals that first had an opportunity to consider the trial for publication. Only one (1%) of 99 retrospectively registered trials acknowledged late registration in its publication, attributing the delay to principal investigator oversight and offering access to the original study protocol upon request [27].

**Table 4:** Illustrative examples of prospective trial registration, retrospective trial registration, and retrospective registration after initial primary outcome ascertainment

Reference	Registration no.	Registration date	Enrollment start	Registration delay	Registered primary outcome (time frame)	Registration timing
*J Am Soc Nephrol*. 2010 Jun;21(6):1052–61.	NCT00426153	1/22/2007	1/31/2007	N/A	Percentage change in liver volume (12 months)	Prospective
*J Allergy Clin Immunol*. 2015 Mar;135(3):670–5.e3.	NTR2205	2/8/2010	1/1/2010	1 month	Induced sputum neutrophil and eosinophil percentage counts (9 weeks)	Retrospective
*J Allergy Clin Immunol*. 2015 Apr;135(4):922–29.e6.	NCT02024659	12/27/2013	9/30/2010	39 months	Nasal polyp size (2 weeks)	Retrospective (after initial primary outcome ascertainment)

Journals did not differ significantly in their rates of overall prospective registration (*p* = 0.21), but did differ in their rates of registration before initial primary outcome ascertainment (*p* = 0.004) (Table 2). Trials involving industry funding, enrollment sites in the US, and assessing drugs, devices, or vaccines/biologicals each had higher rates of prospective registration as compared to those without industry funding, enrolling at only non-US sites, and assessing non-regulated interventions (Table 3).

### Primary outcome concordance

Among the 439 registered trials, 15 (3.4%) failed to register a primary outcome at initial registration, though 14 of these 15 published a primary outcome. Twenty-six trials, nearly all of which (*n* = 25; 96%) registered a primary outcome at initial registration, did not designate one in their publications. Of 413 registered trials designating at least one primary outcome in their publications, 60 % (*n* = 249) published primary outcomes that were non-discordant from those specified at initial registration. Twenty-six percent (*n* = 109) published primary outcomes discordant from those initially registered. Seventy-eight (72%) of these 109 discrepancies were based on either the number or definition of primary outcomes, whereas 31 (28%) were based on the specified time frame of primary outcome ascertainment. The remaining 13% (55 of 413) registered initial primary outcomes that were too poorly specified to permit comparison with published outcomes. Among the 346 trials registered first at ClinicalTrials.gov, 19 (5%) trials listed original primary outcome measures that were submitted at least 30 days subsequent to the reported registration date. Seven (37%) of these 19 involved trials whose registration was already retrospective.

Among the 249 trials reporting discordant published and registered primary outcomes, 80% (*n* = 198) were registered prospectively; 20% (51 of 249) were retrospectively registered. Neither overall prospective registration (RR = 1.06, 95% CI = 0.95–1.18; *p* = 0.31) nor registration prior to initial primary outcome ascertainment (RR = 1.05, 95% CI = 0.96–1.14; *p* = 0.29) was associated with non-discordance between registered and published primary outcomes. Even so, just one of seven trials determined to have been registered after their primary completion date published outcomes non-discordant with those initially registered, despite the significant delay in registration.

### Favorability of trial findings

Among the 486 trials in our sample, 425 (87%) reported primary outcome findings from which inferences about the statistical significance of reported outcomes could be drawn; 61 trials (13%) were non-inferential, including descriptive or single-arm studies without a specified comparator, and could not be judged accordingly. Sixty-six percent (*n* = 282) of the 425 inferential trials reported favorable primary outcome findings. Of 143 (34%) trials reporting unfavorable primary outcome findings, most (*n* = 135; 94%) reported findings that were not significant, while eight (6%) reported negative findings. Unregistered trials were more likely to report favorable findings (31 of 35; 89%) than were registered trials (251 of 390; 64%) (RR = 1.38, 95% CI = 1.20–1.58; *p* = 0.004), irrespective of registration timing. Favorable findings reporting appeared to be more frequent among trials potentially vulnerable to unaccounted primary outcome modifications (73 of 96; 76%), which included those that were unregistered and those registered after initial primary outcome ascertainment, compared to those registered prior to initial primary outcome ascertainment (206 of 321; 64%), but our findings did not reach statistical significance (RR = 1.18, 95% CI = 1.03–1.36; *p* = 0.03).

## Discussion

Our study of clinical trials recently published in ten high-impact specialty society journals, all requiring trial registration, found that 10% of published trials were unregistered. Moreover, among registered trials, nearly one quarter were registered retrospectively. Of these, more than two thirds, or 15% of all registered trials, were registered after potential observation of primary outcome data, affording opportunity for unaccounted protocol modifications based on potential premature analyses of observed primary outcome data. Irrespective of registration timing, post-registration modifications to primary outcomes were frequent, as 26% of trials published primary outcomes that differed from those specified at initial registration. Finally, unregistered trials reported favorable findings at a higher rate than trials that had registered. The publication of unregistered trials and trials registered after initial primary outcome ascertainment raises concerns about selective reporting and the integrity of reported outcomes, as these trials are vulnerable to potential changes obscured from public record.

Despite policies to improve registration rates [25, 28, 29], publication of unregistered trials persists. Our study demonstrates that even the highest-impact journals associated with US professional medical societies publish unregistered trials, albeit some more frequently than others. Consistent with earlier studies [7–13], our findings suggest that, more than a decade since implementation of policies designed to promote universal registration, continued efforts are needed to ensure that all trials are registered, even among those that are published. Registration was more frequent among trials assessing FDA-regulated interventions as compared to trials evaluating non-regulated interventions, such as behavioral and procedural interventions, as prior research has suggested [10]. Unlike trials of FDA-regulated interventions, trials evaluating non-regulated interventions are not subject to legal requirements, which may explain observed differences in registration rates. Inconsistencies between legal and ICMJE registration policies may in fact serve as a potential source of confusion to investigators. We additionally noted higher registration rates among larger trials and those receiving industry support. As each of the specialty society journals we assessed requires trial registration, our results indicate that some journals do not consistently adhere to their own registration policies. Prior work indicates that journals may in fact relax their own registration requirements for various reasons, including reluctance to penalize otherwise sound research, apprehension about losing manuscripts to rival journals, and misconceptions about the applicability of registration policies [19]. Regardless of the rationale, publication of unregistered trials risks dissemination of trials lacking accountability and potentially influenced by selective reporting. Our study and prior work examining cardiovascular clinical trials demonstrate that unregistered trials more frequently report favorable findings [30], though a recent study examining a large sample of unselected trials found only a marginal association [31]. Nevertheless, stricter adherence to registration policies may help prevent the publication of trials that are selectively reporting results, biasing the medical literature.

While registering trials can help mitigate selective reporting, registration must occur prospectively, in accordance with ICMJE policy, to effectually detect and deter biased reporting. Despite the importance of prospective registration, nearly one in four trials in our sample was published despite having been registered retrospectively. Furthermore, the majority of late registrations were delayed to such a degree after enrollment of the trials’ first participants that it could have permitted investigators the opportunity to amend primary outcomes after conducting interim analyses. For trials registered after ascertainment of outcomes, it is nearly impossible to ascertain the degree to which published reports diverge from the original protocol given the potential for modifications occurring covertly pre-registration. While the frequency of post-registration outcome modifications does not appear to depend on the timeliness of trial registration, we cannot comment on the frequency and effects of pre-registration protocol modifications beyond identifying situations in which they could have potentially occurred.

Our findings are consistent with prior research that prospective registration is more frequent among certain trial types, including those involving FDA-regulated interventions and those receiving industry support [8, 15]. Compared with existing studies [8, 15, 32], however, retrospective registration was overall less frequent in our sample. Notwithstanding the possibility that specialty society journals are in better overall adherence, there are several methodological explanations for this observation, including utilization of each trial’s earliest registration record, which is not always reported in publications, application of a 30-day grace period between enrollment initiation and registration, and our conservative treatment of month-based reporting of enrollment start dates to ensure that true prospective registrations were not misclassified. Only one prior study has assessed the timeliness of registration as it relates to its potential effect on reported outcomes, specifically within the context of the six highest-impact general medical journals [15]. While late registration was less frequent among specialty society journals as compared to the general medical journals assessed in the prior study (23% vs. 28%), we observed a higher proportion of late registrations that potentially permitted an opportunity for outcome modification informed by potential interim analyses (15% vs. 8%). Our findings are further consistent with prior research on selective outcome reporting, indicating that discrepancies between registered and published primary outcomes persist among clinical trials [33–35]. A systematic review of studies comparing protocol or registry entries against publications of randomized controlled trials found that 4–50% of trial reports contained evidence of selective reporting involving primary outcomes, a range in line with our observation that 26% of trials published primary outcomes that differed from those specified at initial registration [36]. No prior study has examined the association between registration timeliness and selective reporting in terms of discordance between registered and published primary outcomes. Our findings suggest that the frequency of primary outcome discordance is not different between prospectively and retrospectively registered trials.

### Implications of study findings

Because journals control the dissemination of research, they are well positioned to help ensure the integrity of published material, which includes adequate and prospective registration of published trials. Specialty society journals, in particular, bear a significant responsibility to this end, as they publish trials that are of great interest and potential influence to their targeted clinical readerships. As part of the peer-review process, journals generally require the disclosure of trial registration information, though discrepancies between registered and reported material do not appear to influence the decision to accept or reject manuscripts [37], suggesting that editors may not scrutinize or may choose to disregard discrepancies. If oversight is, in fact, the driver, greater attention paid to trial registration during editorial review may reduce the rate at which potentially biased trials are published, including those that are unregistered or retrospectively registered.

However, while ICMJE policy advocates barring retrospectively registered trials from publication, it acknowledges that editors may judge for themselves the circumstances surrounding late registration and its potential bearing on reported outcomes [25]. Accordingly, our findings may instead stem from editors deliberately choosing to publish non-compliant trials, which they may do for reasons suggested previously [19]. A survey of editors from journals endorsing ICMJE guidelines found that two thirds would consider publication of retrospectively registered trials, though just 13% indicated that consideration would be situation-dependent [18]. For journal editors weighing the decision to publish such trials, ascertaining whether registration was sufficiently delayed to have potentially biased the reported results may help guide decisions regarding appropriate exceptions. The significance of study findings should be carefully evaluated in the decision to accept or reject given the potential for bias that exists among unregistered or retrospectively registered trials. If journals elect to move forward with publishing these trials, steps should be taken to ensure that original trial protocols, approved by and obtained directly from Institutional Review Boards, are made publicly available. Additionally, as ICMJE policy suggests, publication of non-compliant trials should be accompanied by published statements explaining why registration did not occur or was delayed and, further, why journal editors nonetheless judged the trial fit for publication [25]. Just one retrospectively registered trial in our sample addressed its delayed registration, offering to make available its original protocol upon request. While routine posting of original protocols for all trials, regardless of registration compliance, may mitigate concerns regarding biased reporting, such practices are infrequent [38]. Among journals in our sample, only the *Journal of Clinical Oncology* requires submission and publication of trial protocols, albeit only for phase II and III trials [39].

### Limitations

Our study has limitations. First, the ICMJE definition of a clinical trial is subject to interpretation, particularly in terms of what constitutes a “health-related intervention” and a “health outcome.” ICMJE adopted an expanded clinical trial definition in 2007 clarifying the scope of these terms [40]. Nevertheless, confusion regarding the applicability of registration requirements for interventional clinical studies may exist among investigators and journals editors. While ICMJE believes that investigators should err towards prospectively registering all interventional studies of human subjects in cases of uncertainty [40], subjectivity in classifying studies as “clinical trials” may have influenced our observed frequency of unregistered trials, particularly in cases where the applicability of the ICMJE definition may not be patent. Second, our analysis does not represent a perfect audit of ICMJE registration policy, given our concession of a 30-day grace period and exclusion of phase I studies. Nevertheless, we aimed to capture the spirit of the policy rather than the strict letter of the law to account for potential flexibility on the part of journals in the case of minimally delayed registrations. Third, our sample by design comprised a group of clinical trials recently published in select high-impact specialty society journals; accordingly, our findings may not be representative of overall trial registration patterns or of all specialty society journals. Nevertheless, we selected the highest-impact specialty journals, the most prestigious in their respective fields, which are expected to adhere to the highest standards of trial registration practices. Fourth, our cross-sectional analysis did not examine potential improvements in trial registration within journals over time nor account for the fact that journals may have adopted the ICMJE’s registration policy at different time points since its implementation. Even so, the earliest trials in our sample were published in January 2010, nearly half a decade since the policy went into effect, with 89% of sampled trials being published in a 3-year span since 2013. Finally, our study only assessed frequency of modifications to primary outcomes, though selective reporting may manifest through post-registration protocol modifications to other elements of trial design, including secondary outcomes and sample size, which we did not examine. Moreover, we were only able to comment on the possibility of retrospective registration to invite unaccounted interim analyses or pre-registration protocol modifications and not on whether such analyses or modifications actually occurred. Such information could only be ascertained through examination of original trial protocols, which are often unavailable and lack complete information [38]. Additionally, how informative interim analyses are, in some cases, depends on the trial’s experience of participant accrual, details of which are also generally not readily accessible.

## Conclusions

Our large study of clinical trials published in ten high-impact specialty society journals demonstrates that registration of trials continues to fall short of the ICMJE’s standards necessary to ensure a complete and unbiased evidence base. Ten percent of published trials were unregistered. Moreover, nearly a quarter of registered trials were registered late, the majority of these after potential observation of primary outcome data, potentially affording investigators the chance to implement modifications potentially informed by collected data. Unregistered trials reported favorable study findings at a higher rate than registered trials, raising concerns that lack of accountability may exert undue influence on reported findings. While journals should generally avoid publishing improperly registered trials, exceptions should be acknowledged, justified, and furthermore accompanied by an evaluation and public posting of the study’s original protocol. Greater adherence to the ICMJE’s prospective trial registration policy may help reduce the publication of studies failing to meet proper standards and improve the integrity of published trial findings.

## Additional file

Additional file 1: Study protocol. This file contains the original study protocol in addition to a listing of protocol amendments. (DOCX 156 kb)

## Abbreviations

CI: Confidence interval
FDA: US Food and Drug Administration
ICMJE: International Committee of Medical Journal Editors
ICTRP: International Clinical Trials Registry Platform
RR: Relative risk
WHO: World Health Organization
