# Supplementary material for: Adherence to the International Committee of Medical Journal Editors’ (ICMJE) prospective registration policy and implications for outcome integrity: a cross-sectional analysis of trials published in high-impact specialty society journals
Source: Trials. 2018 Aug 23;19:448. doi: 10.1186/s13063-018-2825-y (PMC6106722; doi:10.1186/s13063-018-2825-y)
Supplement: Supplementary file 1 — Study protocol. This file contains the original study protocol in addition to a listing of protocol amendments. (DOCX 156 kb) [file 13063_2018_2825_MOESM1_ESM.docx]

**Prospective Registration among Trials Published in High-Impact Specialty Society Journals**

**STUDY PROTOCOL**

**I. Specific Aims**

1. To determine the frequency of registration among clinical trials published in high-impact specialty journals
2. To characterize rates of “prospective” versus “retrospective” registration of clinical trials published in these journals
3. Among retrospectively-registered trials, to identify, where possible, the proportion for which registration occurred after initial ascertainment of the primary outcome

**II. Methods**

**IIa. *Definitions***

The ICMJE defines a clinical trial as “any research project that prospectively assigns people or a group of people to an intervention, with or without concurrent comparison or control groups, to study the cause-and-effect relationship between a health-related intervention and a health outcome” (ICMJE, 2007). “Prospectively” registered trials, as defined by the ICMJE, are those registered on or prior to the date of enrollment of the trial’s first participant. Trials registered after enrollment initiation are considered to have been registered “retrospectively.” According to ICMJE registration policy, trials that began recruiting patients on or after July 1, 2005 must be registered prospectively for consideration for publication. Trials that started enrollment prior to the date of policy enactment are considered by the ICJME to have been “ongoing” if data collection and analysis were pending; these trials must also register prior to manuscript submission, though retrospective registration is permissible. The ICMJE accepts registration of trials in ClinicalTrials.gov in addition to any of the primary registries that comprise the WHO International Clinical Trials Registry Platform (ICTRP).

**IIb. *Sampling strategy***

We will compile a list of all medical professional organizations in the US as registered with the American Board of Medical Specialties (American Board of Medical Specialties, 2016). We will then identify the medical journal(s) associated with each of these specialties and their 2015 impact factors as listed on Thomson Reuters InCites™ Journal Citation Reports (Thomson Reuters, 2015). We will search for additional journals using SCImago Journal & Country Rank listings and add to our list any that are associated with a US-based medical specialty organization (Scimago Lab, 2016). We will then rank these specialty society journals based on impact factor and select the ten highest-ranked journals. We will exclude journals that pertain to general practice, as our focus is studying specialty journals. Finally, for each journal in our sample, we will confirm endorsement of ICMJE policies on clinical trial registration by reviewing either the journal’s website for a statement indicating a trial registration policy in accordance with the ICMJE’s or verifying listing of the journal on the ICMJE’s website.

Clinical trials will be identified by review of the Abstract and/or Methods section of original research reports (including brief reports but not research letters/correspondences) for statements indicating that the study described is, per the ICMJE definition, a health-related prospective interventional design involving human subjects. We will limit our sample to primary publications of trials (identified as those reporting the results of the trial’s primary outcome at the time point, if applicable, described within the publication’s Introduction and/or Methods section as part of the trial’s main analysis). Publications will be reviewed in the order listed within each journal issue’s Table of Contents. Within each specialty journal, we will sample the 50 most recently published primary publications of clinical trials, restricting our sample to trials published between January 2010 and December 2015. If a journal has not published at least 50 trials within this 6-year period, we will use the number published during this timeframe as our sample size for that journal. We will exclude publications of Phase I trials, as these studies are exempt from federal registration requirements and typically have minimal impact on clinical practice. We will additionally exclude publications of trials that began enrollment prior to July 2005, the date of ICMJE policy enactment, since trials that began enrollment prior to this are likely to have been retrospectively registered. If an article reports on more than 1 trial, we will include all trials described therein.

**IIc. *Data collection***

Overall and within each specialty journal, we will identify the following outcomes:

- Proportion of trials registered in any ICMJE-accepted registry
- Proportion of trials registered prospectively vs. retrospectively
- Among trials registered retrospectively, the proportion registered after initial ascertainment of the primary outcome

From trial publications, we will collect the following data elements: journal, date of online publication, intervention type (drug vs. vaccine/biologic vs. device vs. other), allocation mechanism (randomized vs. non-randomized), date of enrollment initiation (if available), and the registration number(s) associated with the trial(s) described therein. While we expect that most trials will be registered in a single registry, some may be registered in additional trial registries not reported in the publication. Because the reported registration may not necessarily correspond to a trial’s first registration, we will search the WHO ICRTP platform using the reported registration identifier in addition to reviewing the reported registration record for alternate registration numbers. Among all registrations for a trial, we will record the earliest registration, which we denote as the primary, and make note of all subsequent additional. We will further verify that the trial was registered within a registry accepted by the ICMJE. If a registration number is not reported in the publication, we will perform a search of all ICMJE-accepted registries (via the WHO ICTRP platform) using search terms pertaining to the intervention name, first author, senior author, sponsor, and target sample size before concluding that the published trial is unregistered.

From the primary trial registry, we will record the registration date and the date of enrollment initiation. Using this information, we will determine whether registration occurred prospectively or retrospectively. Among trials initially registered on ClinicalTrials.gov, we will also collect the date corresponding to submission of the original primary outcome measure. For enrollment initiation dates reported as only a month and year, we will consider the last day of the corresponding month as the date in question (i.e. September 2012 would be converted to September 30, 2012) so as to remain conservative in classifying registrations as retrospective.

Among trials deduced to have been registered retrospectively, we will next establish whether registration might have occurred after primary outcome ascertainment by comparing the trial’s date of registration against the date on which primary outcome ascertainment would have occurred for the trial’s first participant given the registered enrollment initiation date. Among all trials, we will also compare the original primary outcome(s) as reported in the registry record with the outcome(s) reported within the trial’s publication to note differences in registered and published primary endpoints.

Additional information to be extracted from the primary registry record includes anticipated enrollment, study phase, study location(s) (US/Canada only vs. US/Canada and international vs. international only), funding source (industry vs. NIH vs. other government entity vs. academic institution vs. other), and the primary completion date (i.e. date of final data collection for the primary outcome measure).

For trials that have been registered retrospectively, we will review the corresponding publication for a statement acknowledging or explaining the delay by searching for the following terms: “registration,” “retrospective,” and “delay”.

**IId. *Data Validation***

Two investigators will work simultaneously to perform a uniform fraction of abstractions across journals. Upon completion of data abstraction for all publications in the sample, we will randomly select 10% of trials for validation. Each data abstractor will validate the other's randomly selected abstractions in proportion to the number that the other abstracted. We will calculate a measure of inter-rate agreement. A third investigator with expertise in clinical trial design and reporting will adjudicate discrepancies and determine whether systematic disagreements warrant re-abstraction of particular data elements. For trials designated as “unregistered,” a third investigator will independently review the appropriateness of their inclusion in the sample and subsequently perform an additional search of the WHO ICRTP platform to ensure that they are indeed unregistered.

**IIe. *Statistical analysis***

Our primary analysis will include a presentation of descriptive statistics summarizing the proportion of trials registered and the proportion registered prospectively/retrospectively, overall, and stratified by specialty journal. Significant differences across journals will be assessed using a chi-squared test. Among trials registered retrospectively, we will present the proportion registered after initial ascertainment of the primary outcome. We will also report the proportion of trials with differences between the originally registered and published primary outcome measures, overall, and stratified by registration status. Association between registration status and primary endpoint consistency will be characterized using a chi-squared test.

We will additionally conduct sensitivity analyses around prospective/retrospective registration status determination using the published enrollment initiation date (instead of the registered enrollment initiation date) and report the above outcomes in parallel using this approach.

Secondary statistical analyses will include comparisons of prospective registrations by trial characteristics, including intervention type, FDA regulation status, funding source, enrollment (categorized based on median split), and location. We will perform tests of association between study characteristics and prospective/retrospective registration status by fitting a series of bivariate models with trials as the unit of observation and robust standard errors clustering by journal. Analyses will be conducted using JMP version -- (SAS Institute Inc).

**STUDY PROTOCOL AMENDMENTS**

| **Protocol Section** | **Amendment** | **Rationale** |
| --- | --- | --- |
| I. Specific Aims | d. To determine characteristics associated with timely registration | This aim was described in the Methods section of the original protocol, but not included previously as an objective. |
| I. Specific Aims | e. To assess the frequency at which trials publish primary endpoints concordant with those initially registered | This aim was described in the Methods section of the original protocol, but not included previously as an objective. |
| I. Specific Aims | f. To evaluate whether trials lacking accountability (e.g. unregistered trials and those registered after initial primary outcome ascertainment) are more likely to report favorable findings | This aim was not included in the original protocol, but was added to the study after data collection was completed though before relevant analyses were conducted. Study results were classified in a blinded fashion with regard to registration status according to the scheme outlined in the “Methods, Main Outcome Measures” section of the manuscript. |
| IIc. Data collection | The following additional data elements were collected: manuscript submission date | This data element was not included in the original protocol but later collected to determine the number of trials registering after submission to the publishing journal. |
| IIc. Data collection | For all trials for which we could not locate a registration record, we additionally emailed corresponding authors to inquire about registration information before concluding that the trial was unregistered. | This additional search strategy was not included in the original protocol, but added later in order to increase the specificity of our ‘unregistered’ determinations. |
| IIc. Data collection | For all trials for which we could not locate a registration record, a third authorperformed searches on NIH REPORTER using grant identifiers where available. | This additional search strategy was not included in the original protocol, but added later in order to increase the specificity of our ‘unregistered’ determinations. |
| IIc. Data collection | When information on enrollment, phase, location, and funding source was missing from trial registries or the trial was unregistered, this information was collected from trial publications. | This statement was not included in the original protocol but added to account for potentially missing data from trial registration records. |
| IIc. Data collection | In determining whether registration was prospective vs. retrospective, we allowed a 30-day grace period between enrollment start and registration. | This 30-day window was not specified in the original protocol, but later determined to be most appropriate (prior to analyses) to account for potential journal flexibility in enforcing ICMJE policy among trials whose registration may have only been slightly delayed. |
| IIc. Data collection | The specific scheme for comparing primary outcome measures was not described in the original protocol. As outlined in the manuscript, we classified published-registered primary outcome pairs as follows: “We classified registered-published primary outcome pairs as discordant if they differed in any of the following: number of primary outcomes, definition(s) of primary outcomes, or specified time frame(s) for outcome(s) ascertainment. If no discrepancies were noted in these three domains, pairs were classified as concordant. We noted cases where registered endpoints were too poorly specified (e.g. vague study of “efficacy of intervention”) to permit comparison.” | The specific details of our classification scheme were not included in the original protocol, but were decided on prior to any analysis of the data. Outcome pairs were classified in a blinded fashion with regards to registration status. |
| IIc. Data Collection | We determined registration timeliness using the registry enrollment start date rather than the published enrollment start date. | This detail was not specified in the original protocol, but was decided prior to data collection, as we anticipated, based on experience with the medical literature, that publications may not consistently report enrollment start dates. We also collected publication enrollment start dates when available for the purposes of a sensitivity analysis (specified in IIe. Statistical Analysis of the protocol and reported in Appendix 2 of the study supplement) around registration timeliness. |
| IIc. Data collection | In cases where the primary endpoint(s) included multiple time frames, we used the shortest time frame specified in the registry in our determinations of whether registration might have occurred after initial primary outcome ascertainment. If time frame information was not specified in the registry, we used the shortest time frame reported in the publication. | This detail was not specified in the original protocol, but was decided prior to data collection to ensure consistent determinations of whether the primary endpoint might have been ascertained prior to trial registration among retrospectively registered trials (in cases where such a determination was possible). |
| IIe. Statistical Analysis | Our statistical analysis plan as specified in the original protocol includes the following amendments, underlined below:  “Our primary analysis will include a presentation of descriptive statistics summarizing the proportion of trials registered and the proportion registered prospectively/retrospectively, overall, and stratified by specialty journal and trial characteristics. Trial characteristics will include and will be coded as follows: intervention type (drug/device/biological vs. non-drug/device/biological, funding source (industry vs. non-industry), enrollment (categorized based on median split), randomization (yes/no) and location (US vs. non-US). Significant differences across journals and trial characteristics will be assessed using a chi-squared test. Among trials registered retrospectively, we will present the proportion registered after initial ascertainment of the primary outcome. Significant differences across journals and trial characteristics will be assessed using chi-squared testing. We will also report the proportion of trials with differences between the originally registered and published primary outcome measures, overall, and stratified by registration status, journal, and trial characteristics. Association between registration status, journal, and trial characteristics and primary endpoint consistency will be characterized using a chi-squared test. We will additionally conduct sensitivity analyses around prospective/retrospective registration status determination using the published enrollment initiation date (instead of the registered enrollment initiation date) ~~and report the above outcomes in parallel using this approach~~. *[The sensitivity analysis using published enrollment start will be reported in Appendix 2 of the study supplement*]  [*Note that* *portions of the next paragraph are redundant with the above]* Secondary statistical analyses will include comparisons of prospective registrations by trial characteristics, including intervention type (drug/device/biological vs. non-drug/device/biological), ~~FDA regulation status~~, funding source (industry vs. non-industry), enrollment (categorized based on median split), randomization (yes/no) and location (US vs. non-US). We will perform tests of association between study characteristics and prospective/retrospective registration status by using chi-squared testing ~~fitting a series of bivariate models with trials as the unit of observation and robust standard errors clustering by journal~~. We will additionally determine the proportion of trials reporting favorable results, overall, and stratified by journal, registration timeliness, and trial characteristics. We will use Chi-squared testing to assess differences in study results by journal, trial characteristics, and timeliness of registration. Analyses will be conducted using JMP version -- (SAS Institute Inc). | The statistical analysis plan as explicitly outlined in the original trial protocol was not sufficiently detailed. The amendments made to the statistical analysis plan, specifically with regards to specific testing, were made prior to analysis of the data. |
